# Vasopressin Proves Es-sense-tial: Vasopressin and the Modulation of Sensory Processing in Mammals

**DOI:** 10.3389/fendo.2015.00005

**Published:** 2015-02-05

**Authors:** Janet K. Bester-Meredith, Alexandria P. Fancher, Grace E. Mammarella

**Affiliations:** ^1^Department of Biology, Seattle Pacific University, Seattle, WA, USA

**Keywords:** vasopressin, sensory, olfaction, social behavior, hearing, gustation, social learning

## Abstract

As mammals develop, they encounter increasing social complexity in the surrounding world. In order to survive, mammals must show appropriate behaviors toward their mates, offspring, and same-sex conspecifics. Although the behavioral effects of the neuropeptide arginine vasopressin (AVP) have been studied in a variety of social contexts, the effects of this neuropeptide on multimodal sensory processing have received less attention. AVP is widely distributed through sensory regions of the brain and has been demonstrated to modulate olfactory, auditory, gustatory, and visual processing. Here, we review the evidence linking AVP to the processing of social stimuli in sensory regions of the brain and explore how sensory processing can shape behavioral responses to these stimuli. In addition, we address the interplay between hormonal and neural AVP in regulating sensory processing of social cues. Because AVP pathways show plasticity during development, early life experiences may shape life-long processing of sensory information. Furthermore, disorders of social behavior such as autism and schizophrenia that have been linked with AVP also have been linked with dysfunctions in sensory processing. Together, these studies suggest that AVP’s diversity of effects on social behavior across a variety of mammalian species may result from the effects of this neuropeptide on sensory processing.

## Introduction

In social species, survival depends on navigating complex social cues. An individual must be able to use sensory cues to distinguish between familiar and unfamiliar individuals. These sensory cues must then be filtered in a way that directs the animal to make an appropriate behavioral response. Although interpretation of sensory cues is influenced by many neural pathways, the neuropeptide arginine vasopressin (AVP) appears to be critical for making these key social distinctions in mammals. This neuropeptide has been associated with pair bonding, aggression, parental care, social memory formation, and stress responses in multiple species of rodents [reviewed in Ref. (1)], possibly due to its broader role in integrating sensory input. In addition, the distribution of AVP and its receptors changes throughout the lifespan. During development, AVP immunoreactivity and receptors show remarkable plasticity in response to environmental influences, such as the quality and quantity of interactions with peers and parents (2–10). Therefore, early developmental experiences may shape how an animal interprets sensory cues related to social behavior throughout its lifespan.

The focus of this article is to review evidence that links AVP with the processing of sensory information and to explore whether this alteration of sensory processing leads to diverse behavioral effects across different mammalian species. This paper describes the distribution of AVP and its receptors within sensory organs and within brain areas that receive direct sensory input. We also describe anatomical pathways containing AVP and its receptors that connect the primary sensory cortices with brain areas that process social cues and that direct complex forms of social behavior. We are proposing that the sex-specificity and species-specificity of AVP effects on behavior in mammals result from the variation in the pattern of distribution of AVP and its receptors in sensory pathways. The convergence of sensory input with vasopressin pathways that travel both within the brain and outside of the brain suggests that the effects of AVP on behavior may be mediated by both central pathways that alter the valence of social stimuli and peripheral pathways that alter physiological responses to social stimuli.

## Background

### Sexual dimorphism in AVP: Differences between parvocellular and magnocellular neurons

Arginine vasopressin has been localized in parvocellular and magnocellular neurons that are widely distributed in the central nervous system (11). AVP within a pathway that originates in the medial amygdala (MA) and bed nucleus of the stria terminalis (BNST) and projects to the lateral septum (LS) has been associated with complex social behavior in mammals [reviewed in Ref. (1)]. Early exploration into the association between this pathway and sex-specific patterns of social behavior originated with the identification of sexual dimorphism in AVP immunohistochemistry. In rats (*Rattus rattus*), prairie voles (*Microtus ochrogaster*), meadow voles (*Microtus pennsylvanicus*), and CD1 mice (*Mus musculus*), males show more AVP-immunoreactive (AVP-ir) staining in the BNST and its projections to the LS than do females (12–14). The sexual dimorphism in AVP appears to be testosterone-dependent because castration reduces AVP immunoreactivity in male rats and testosterone implants increase AVP immunoreactivity in female rats (15–17). Although AVP is found only in mammals, similar sexual dimorphism has been observed in the homologous arginine vasotocin (AVT) pathways of birds, reptiles, and amphibians (18). Although the role of AVT in regulating sensorimotor processing in the amphibian *Taricha granulosa* has been explored elsewhere (19), the role of AVP in regulating sensory processing in mammals has received less attention.

Because AVP immunoreactivity is more pronounced in the male brain, it has been suggested that AVP may regulate male social behavior, and that oxytocin may serve a similar role in the female brain. Early evidence supported the contention that AVP was a key regulator of male social behavior during pair-bonding and aggressive encounters, but more recent evidence also implicates AVP in regulating female social behavior (9, 20–30). The role of AVP in regulating species-specific social behavior in both males and females in different ways in a variety of mammalian species suggests that this neuropeptide serves a broader function in behavioral regulation.

Although behavioral studies usually focus on AVP in the parvocellular pathway described above, AVP produced within magnocellular neurons of the hypothalamus also regulates physiological functions that may indirectly influence an animal’s behavioral responses to social stimuli. After its release from the posterior pituitary gland, AVP also enters into the bloodstream where it produces hormonal effects on a variety of peripheral tissues. The magnocellular neurons of the paraventricular nucleus (PVN) of the hypothalamus and of the supraoptic nucleus (SON) appear to be the main sites of AVP production in this pathway and can be distinguished from the parvocellular neurons by measuring their voltage-gated currents (31). Within the bloodstream, AVP is more commonly known as anti-diuretic hormone (ADH), a hormone that increases blood pressure and decreases ion concentrations within the blood by lowering the amount of water that is excreted in urine. AVP also modulates cardiac function by increasing activity of the sympathetic neurons that innervate the heart and decreasing activity of parasympathetic neurons [reviewed in Ref. (32)]. Although the changes in blood pressure and heart rate produced by AVP in response to activation of magnocellular neurons are often overlooked in studies of social behavior, the coupling of these effects with central effects can be critical for normal emotional responses (33). Appropriate responses to social stimuli may be more likely to occur when alterations in heart rate and blood pressure create an optimal level of arousal that allows the animal to focus on subtle social cues.

### Distribution of three types of AVP receptors

Arginine vasopressin produces different effects within the central and peripheral nervous systems because it binds to three main categories of receptors: V1a, V1b, and V2 receptors. Although V2 receptors and both subtypes of V1 receptors are found within the peripheral nervous system, only V1 receptors are found within the central nervous system. Despite the early suggestion that binding of AVP to V1a receptors was responsible for all of the behavioral effects of this neuropeptide, more recent evidence using receptor knockouts indicates that central V1b receptors also are critical for social behavior [(1); for review of V1b receptors, see Ref. (34, 35); for review of V1a receptors, see Ref. (36–38)]. Adding an additional layer of complexity to our understanding of the central effects of AVP is the observation that AVP can bind to oxytocin receptors and that oxytocin may act as an agonist at AVP receptors because of the similarities between the structures of these peptides and their G-protein linked receptors [(39); reviewed in Ref. (40)]. However, the impact of this potential peptide cross-reactivity on social behavior is unclear as it has not been well studied.

Although V1a receptor distribution often varies between closely related species with different mating systems [e.g., Ref. (41–44)], consistent patterns of differences between monogamous and non-monogamous species have not yet been identified. Monogamous California mice (*Peromyscus californicus*) show elevated V1a receptor binding in the LS in comparison to non-monogamous white-footed mice (*Peromyscus leucopus*), but studies in voles have found an opposite pattern with higher V1a receptor binding in the non-monogamous species (42, 45, 46). Despite the lack of consistency in patterns of variability across species, social behaviors related to monogamy can be altered using manipulations of AVP within a single species. In prairie voles, a monogamous species, reduction of V1a gene activity using RNA interference reduces partner preference and other behaviors related to monogamy (47). However, the lack of consistency in V1a receptor distribution patterns between closely related species has led to multiple hypotheses about the reasons for these differences. Although it has been suggested that interspecies variation in V1a receptor distribution may result from differences in aggression, different ecological pressures that alter spatial distributions of animals within their habitats, and/or other social pressures, none of these explanations seem to fit all of the existing data (38, 42, 44, 48, 49). Therefore, it seems reasonable to hypothesize that AVP pathways may serve a broader function related to social behavior because of the large amount of sex and species variation in this system.

## Vasopressin and Sensory Signaling

### Vasopressin and olfaction

#### Olfactory brain circuitry: overview

**Figure 1:**
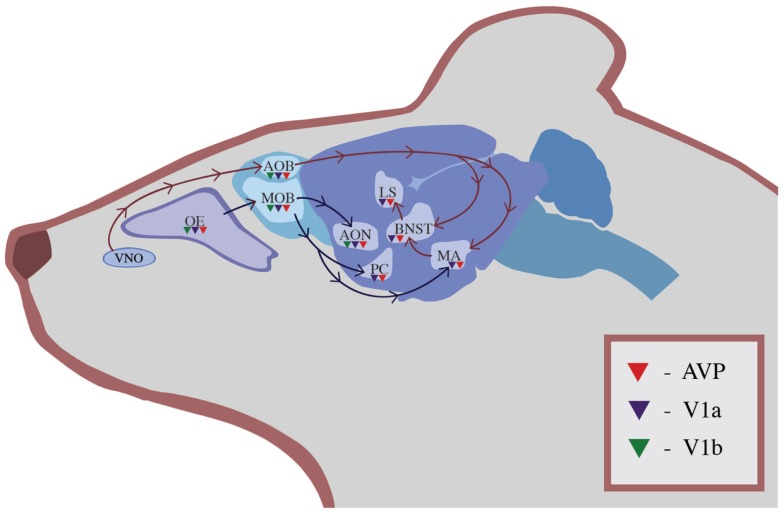
**The distribution of AVP (red triangles), V1a receptors (blue triangles), and V1b receptors (green triangles) in olfactory regions of the rodent brain**. Information in the main olfactory system travels from the OE to the MOB and then is processed in the accessory olfactory nucleus, PC, and MA. Information in the accessory olfactory system travels from the VNO to the AOB and then to the MA, BNST, and LS.

Two separate olfactory systems exist within the rodent brain that process socially relevant olfactory information. Both of these pathways contain AVP and its receptors (Figure 1). In the main olfactory system, input travels from the main olfactory bulb (MOB) to the anterior olfactory nucleus, piriform cortex (PC), and amygdala for additional processing (50). This multi-step pathway brings olfactory information to be processed in multiple areas of the cerebral cortex, including the anterior olfactory nucleus and PC. The anterior olfactory nucleus, a cortical area adjacent to the olfactory bulb that is part of the main olfactory pathway, contains AVP neurons that are activated by the exposure to social odors in rats but not by exposure to other odor cues (51). A parallel system, the accessory olfactory system, brings information from the vomeronasal organ (VNO) into the accessory olfactory bulb (AOB), which innervates the MA, BNST, and cortical amygdala (52). Although it was originally assumed that only the accessory olfactory system processed pheromones and other socially relevant odors, more recent evidence suggests that social information is processed by both pathways (52).

This redundancy allows olfactory input to be processed simultaneously by non-dimorphic brain areas and by sexually dimorphic, AVP-rich areas such as the BNST, amygdala, and LS. For example, in male rats, exposure to the odors of a receptive female held behind a perforated plastic partition leads to Fos expression in the AVP neurons of the MA (53). The presence of this neuronal marker of gene activation in the MA indicates that the odor of the estrous female is driving activation of the MA and the interconnected hypothalamic brain areas that are associated with reproductive behavior. A different pattern of neural activation occurs in the brain of a female exposed to the odor of an estrous female. Similarly, female rough skin newts (*T. granulosa*) that have been treated with testosterone and AVT show male-typical responses to female odors, suggesting that manipulations of AVP and its homologs can alter olfactory processing of social cues. The ability of AVP to lead to differentiated responses to complex social odors would be favored by natural selection because these differentiated responses allow mammals to target their behavioral responses toward other individuals in a way that maximizes individual fitness. However, the specific role of AVP in driving complex behavioral responses to social odors has yet to be elucidated.

Arginine vasopressin alters olfactory processing in rats from the moment that olfactory information enters the brain via the MOB or the AOB, but this processing occurs in a variety of complex ways. In rats, the olfactory bulb contains a population of AVP neurons with V1b receptors that does not project outside of the olfactory bulb and that may regulate their own activity (54). This modulation within the bulb could decrease or increase sensitivity to social odors. Social odors also activate OR37 receptors in the main olfactory system, which provide direct, monosynaptic input into both the magnocellular and parvocellular AVP neurons of the PVN and the SON (55). It is not clear, however, whether activation of these receptors leads to release of AVP into the bloodstream, activation of brain areas associated with social behavior, or both. V1a and V1b receptors within the olfactory bulb are also associated with neurons that transmit information outside of the olfactory bulb, and additional V1a and V1b receptors have been localized in the olfactory epithelium (OE) and targets of the olfactory pathways such as the PC (56–58). The presence of AVP and its receptors in so many olfactory processing areas indicates that AVP is able to modulate olfactory information, as the information is evaluated by areas of the brain that direct behavioral responses.

#### Olfactory brain circuitry: interactions between olfactory pathways

Arginine vasopressin’s influence on olfactory processing extends beyond the main and accessory olfactory systems. Although some processing of social odors appears to occur in a sexually dimorphic way to allow males and females to respond differentially to sexual cues, other processing of social odors follows similar neural pathways in both sexes. In mice, anatomical evidence suggests that olfactory cues may stimulate the non-dimorphic cells of the MA and activate the magnocellular cells of the SON (14). SON or PVN activation has the potential to lead to release of AVP in the bloodstream where it may alter the behavior of the animal by affecting the arousal level of the animal due to changes in blood pressure or autonomic activation. However, even though this pathway is not sexually dimorphic, modulation of the activity of this pathway might occur in a sexually dimorphic way through interactions with other neurotransmitter systems or with AVP in other neural pathways that are sexually dimorphic.

Our understanding of how AVP alters olfactory processing has been extended by the use of functional magnetic resonance imaging (fMRI) in addition to traditional neuroanatomical techniques. In this paradigm, fMRI BOLD responses are used to monitor changes in neural activation. Central administration of a V1a receptor antagonist in conjunction with the presentation of a male intruder increases BOLD responses in the anterior olfactory nucleus and in the infralimbic prefrontal cortex and decreases BOLD responses in the cortical amygdala in dams (27). The finding indicates that blocking AVP receptor binding during a threatening situation alters how olfactory cues are processed within the brain. In addition, the alteration in infralimbic prefrontal cortex activation is intriguing because this brain area has been implicated in the regulation of fear and autonomic responses to olfactory stimuli and projects to the BNST and LS (59). Because the BNST and LS have been associated with maternal aggression in rodents (22, 23), this pattern of neuronal activation may indicate readiness to attack an intruder. Although we are only beginning to understand how complex behavioral responses are integrated across the entire brain during social situations, these findings suggest that AVP’s modulation of olfactory systems may have important implications for an animal’s fitness. When AVP receptors are blocked, the typical fear response to an olfactory threat is diminished.

#### AVP and olfactory processing in Syrian hamsters

Arginine vasopressin also has been demonstrated to play a critical behavioral role in the interpretation of chemical signals during social interactions in Syrian hamsters (*Mesocricetus auratus*). In both male and female Syrian hamsters, infusions of AVP into the BNST, LS, or anterior hypothalamus increase the frequency of flank marking (60–62). V1a receptor binding in targets of this pathway, such as the ventromedial hypothalamus, is downregulated in response to social cues. Male Syrian hamsters who win repeated aggressive encounters show less submissive behavior and greater V1a receptor binding in the ventromedial hypothalamus than socially subjugated males (6). Therefore, social experience during development in Syrian hamsters leads to plasticity in AVP receptor distribution that shapes the production of olfactory signals. Although it is not known whether AVP also alters the interpretation of olfactory signals in this species, social odor preferences in both females and males are eliminated with lesions in brain areas that contain AVP and its receptors (63, 64). This plasticity indicates that social experiences that alter the distribution of AVP or its receptors within the brain may also shape a mammal’s ability to produce and respond to olfactory signals.

Despite parallels between the role of AVP in regulating scent-marking behavior in male and female Syrian hamsters, the social context of AVP release may constrain scent mark production differently in males and females. For example, infusions of AVP or a V1a receptor antagonist into the anterior hypothalamus produce opposite effects in males and females. In female, but not male Syrian hamsters, AVP infusions decrease aggression whereas the V1a receptor antagonist infusions increase aggression (65). Together these results indicate that although AVP may shape the production of olfactory signals like flank marking, the social context of these signals determines how this peptide will influence social behavior. Selection pressures, therefore, can lead to sex differences in how AVP influences the processing of olfactory signals.

#### AVP and social recognition

The role of AVP in regulating the interpretation of olfactory signals has also been investigated in other rodents using various social recognition paradigms [reviewed in Ref. (50, 52, 66–69)]. A typical protocol involves the exposure of a rodent to an unfamiliar juvenile conspecific. After a delay of 30-120 min and infusion of AVP or one of its antagonists, the animal then is returned to a testing arena that contains either the same conspecific, a novel animal, or both [e.g., Ref. (70, 71)]. Exposure to the odor of a familiar animal typically leads to less olfactory exploration than exposure to the odor of a novel animal.

Arginine vasopressin influences social recognition via two separate pathways: one pathway that is contained entirely within the olfactory bulb and a second pathway that is sexually dimorphic and leads to the LS. Infusion of AVP into the olfactory bulb enhances social recognition in male rats (72). Although infusions of a commonly used V1a receptor antagonist in the olfactory bulb does not eliminate social recognition, performance in a social recognition test is impaired in male rats after infusions of OPC-21268, a non-peptide V1 receptor antagonist that diffuses more widely (54, 72). In both sexes, infusion of a V1a receptor antagonist into the LS decreases olfactory exploration of a novel same-sex juvenile rat (73). Although this finding is somewhat surprising because AVP immunoreactivity is lower in the LS in female rats, V1a receptors are more abundant in females (73). Therefore, the lower AVP content of the LS in females may be counteracted by the presence of additional receptors in this brain area. These findings also indicate that activation of AVP neural pathways may be important for social recognition in both sexes despite anatomical differences between the sexes.

Although initial processing of the olfactory cues used in social recognition occurs within the olfactory bulb, more complex processing occurs in sexually dimorphic AVP pathways. Innate variation in the production of AVP and its receptors within these areas leads to individual variation in social recognition. Female mice who were categorized as “high recognizers” because of strong performance in a social recognition test expressed less mRNA for AVP, V1a receptors, and V1b receptors in the lateral amygdala than “low recognizers,” although no differences were identified in the BNST or MA (74). The ability to perform well during a social recognition task appears to result from downregulation of AVP in the lateral amygdala even if AVP in other brain areas like the LS is essential for processing the olfactory cues related to the social recognition task. In addition, in some rodent species, AVP pathways used in social recognition are responsive to changes in the environment that alter gene expression through epigenetic mechanisms that change the packaging of DNA and histones within the nucleus of a cell. For example, environmental influences such as maternal separation have been shown to alter methylation of DNA and to alter AVP gene expression (75). Similarly, administration of a histone deacetylase inhibitor like valproic acid creates epigenetic modifications that can alter olfactory processing. In female mice, valproic acid masculinizes AVP fiber density in the LS and increases the attraction of a female toward same-sex odors (76). This finding suggests that epigenetic mechanisms that are shaped by the prenatal and postnatal environment can alter AVP distribution within the brain and modify an animal’s social behavior by modulating responses to olfactory stimuli.

The effects of central release of AVP on social recognition may be amplified by the simultaneous release of AVP into the bloodstream. During a social recognition test in mice using an intact male mouse as a stimulus animal, AVP is released in the SON as measured by microdialysis (77). Because AVP release from the SON leads to elevation of plasma AVP, this indicates that central activation and peripheral activation of AVP pathways may be linked. Lesions of the MA also disrupt the release of AVP from the SON, suggesting that olfactory processing by the pathway projecting from the MA to the LS may activate release of AVP from the SON (77). This connection between the MA and the magnocellular cells of the SON may modulate aggressive responses while elevating levels of AVP in the bloodstream (14).

Despite compelling data indicating that AVP regulates social recognition in both sexes, elimination of one type of AVP receptor does not block social recognition consistently. In male mice, a null mutation in the V1a or the V1b receptor leads to impairments in social recognition [V1a: (78) and V1b: (79)]. Deficits caused by a null mutation in the V1a receptor can be reversed by re-introducing the V1a receptor into the LS using a viral vector (80). However, other studies have failed to identify any impairment in social recognition in male V1a receptor knockout mice despite mild olfactory impairments (81). Female mice with a null mutation of the V1a receptor also display a normal Bruce effect, which is the loss of a pregnancy after the odor of urine of an unfamiliar male activates the vomeronasal system (82). In contrast, female mice with a null mutation in the V1b receptor do not show a normal Bruce effect, indicating that the V1b receptor may be more critical for this response in females (82). Inconsistencies between studies may result from the involvement of multiple receptor types and neurotransmitter systems in processing socially relevant olfactory signals.

#### Evolutionary significance of olfactory processing

The ability to discriminate between the odors of a mate, offspring, and an unfamiliar individual is important for individuals from social species. The evolutionary significance of olfactory investigation, however, may be compounded by the ability to extract additional complex information from odors. Male rats not only can discriminate between individuals but also avoid the odors of ill conspecifics. This avoidance response requires intact AVP neural pathways because exposure to these odors upregulates mRNA for V1a and V1b receptors in the MA, but does not occur if a rat receives a microinfusion of a V1a or V1b receptor antagonist into the MA (83). Although an increase in c-fos mRNA in the olfactory bulb and BNST indicated that those brain areas were activated by exposure to illness-related odors, V1a or V1b receptor binding was not elevated in these brain areas. Therefore, processing of illness-related odors in the AVP system occurs specifically in the MA, a brain area associated with fear. This example illustrates the idea that AVP pathways may assist with creating a complex emotional and behavioral response to the odor of a conspecific that varies depending on the conspecific’s familiarity, sex, health, age, and other individual characteristics.

### AVP and auditory processing

#### Neural processing of auditory signals in birds, fish, and frogs

Although AVP has most commonly been associated with processing of olfactory signals, AVP also assists with processing of auditory signals. A clear link between auditory processing and a non-mammalian AVP homolog, AVT, has been established in birds, frogs, and fish. For example, pairing male and female zebra finches (*Taeniopygia guttata*) increases AVT mRNA in the PVN and BNST of both sexes, and the magnitude of the increase in mRNA production in males is associated with the quantity of singing behaviors (84). Male zebra fiches that choose to sing to a female behind a wire barrier also have more AVT-immunoreactive neurons in the BNST than non-singers, again indicating a linkage between auditory signal production and social behavior in this species (85). In addition to these correlational linkages between AVT and singing behavior, direct manipulations of AVT using intraventricular infusions also increase singing behavior in female sparrows (*Zonotrichia leucophrys gambelii*; 86). Even though these studies do not indicate whether AVT influences auditory processing, an additional study in Lincoln’s sparrows (*Melospiza lincolnii*) demonstrates that sparrows alter the effort used in song production and show changes in AVT-immunoreactivity depending on the quality of songs to which they are exposed (87).

Evidence linking AVT to auditory processing also has been found in fish and frogs. AVT is present in brain areas responsible for auditory integration in the plainfin midshipman fish (*Porichthys notatus*), a species where males vocalize to attract mates and during nest defense (88, 89). Similarly, AVT is more abundant in auditory processing areas of male bullfrogs (*Rana catesbeiana*) in comparison to female bullfrogs (90). Because calls are only produced by males in this species, this sexual dimorphism may indicate that AVT plays a key role in assessing the salience of auditory signals that are being produced by conspecifics. Manipulations of the AVT system support this idea because AVT infusions change call properties of túngara frogs (*Physalaemus pustulosus*), possibly interfering with communication between the sender and the recipient (91, 92). Similarly, in the gray tree frog (*Hyla versicolor*), auditory cues shape the effects of AVT on calling behavior because AVT only alters call quality when a male is in close proximity to another male (93). Together, these results indicate that AVT modulates calling behavior in response to auditory cues and allows animals to produce calls that are appropriate for a particular social context.

#### Neural processing of auditory signals in mammals

The distribution of AVP and its receptors within brain areas associated with auditory processing has been studied in less detail in mammals, possibly because much of the AVP research has focused on rodents that use olfaction as the primary sense for social assessments. Although it is known that female, but not male, guinea pigs (*Cavia porcellus*) display AVP-ir staining in the auditory brainstem, the function of this sexual dimorphism is unknown (94). In the few rodent species where the functional importance of AVP on vocalization has been studied, a role for both V1a and V1b receptors has been identified. Female mice with a null mutation in the V1b receptor show decreased ultrasonic vocalizations during resident-intruder aggressive encounters (95). Although vocal production is affected, it is not known whether auditory processing also differs between control mice and mice with a null mutation in the V1b receptor. Anatomical studies using singing mice (*Scotinomys teguina* and *Scotinomys xerampelinus*) that vocalize in social contexts have identified the presence of V1a receptors in brain areas used for vocal production and auditory responses (49). In both species of singing mice, V1a receptors are expressed in the medial geniculate nucleus, which is the region of the thalamus that processes auditory information (49). In addition, both species display V1a receptor binding in two brain areas associated with vocalization, the periaqueductal gray and anterior hypothalamus, with more extensive binding in the more vocal species, *S. teguina* (49). These findings are intriguing because they implicate AVP in the give-and-take of information that occurs during social communication. In mammalian species where vocalizations are used in social situations, selection may favor a role for AVP in modulating these signals.

Although the effects of central AVP on auditory processing have not been studied in humans due to methodological limitations, human genetic variation in V1a receptor haplotypes has been linked with auditory processing and communication. The number of repeats in the RS3 microsatellite marker for the V1a receptor has been positively linked with prepulse inhibition, a startle response that is suppressed in individuals with schizophrenia and other disorders of social communication (96, 97). This phenotype has been linked more closely to auditory communication in studies examining the relationship between V1a receptor variation and an individual’s musical aptitude. In families containing either a professional or active amateur musician, the ability to detect structural changes in abstract sounds is linked with RS1 and RS3 microsatellite markers for the V1a receptor (98). Interest in music, another marker of interest in listening to auditory cues, is also associated with V1a receptor distribution in humans: the RS1 haplotype is most strongly associated with listening to music regularly at the present time and the RS3 haplotype is most prominently associated with listening to music regularly throughout the lifespan (99). Although studies using viral vectors to manipulate V1a receptor activity cannot be performed in humans, these correlational results indicate that central processing of auditory signals in humans is modulated by AVP and its receptors.

#### AVP as a hormone and acoustic processing in the ear

In addition to affecting neural processing of auditory signals, AVP in the bloodstream also affects hearing. Peripheral injections of AVP in rats create short-term hearing impairment as measured by evoked auditory brainstem responses to sound (100). In the inner ear, AVP also alters hearing by binding to V2 receptors and reducing the number of aquaporin-2 membrane channels, channels that increase water permeability (101). AVP binding to V2 receptors thus leads to hearing impairment through the accumulation of excess water in the membrane of the endolymphatic sac (102, 103). Because insulin interacts with the signaling pathway that regulates V2 receptors, disruption in this pathway in diabetic patients has been linked to hearing loss in humans (103). Similarly, excess endolymphatic fluid due to excess plasma AVP levels or V2 receptors in the inner ear produces the symptoms of Menière’s disease, a disorder in humans that results in intermittent hearing loss, vertigo, and tinnitus (104–106). Therefore, release of AVP into the bloodstream in response to social or stressful stimuli may have a secondary effect of reducing sensitivity to sound cues.

Although excess AVP in the bloodstream may hamper the ability to detect auditory cues, smaller elevations in plasma AVP may increase recall of auditory information due to increased arousal. Peripheral administration of AVP can enhance performance in learning tasks due to elevation of heart rate, blood pressure, and other sympathetic nervous system activity [reviewed in Ref. (107)]. Administration of AVP via intranasal infusions in healthy, non-depressed elderly humans increases recall of auditory information, possibly due to this increase in arousal (108). Natural variation in AVP in humans also is correlated with performance on auditory learning tasks. In humans with major depression, plasma concentrations of AVP correlate positively with auditory memory as measured by a 10-WLLA test for audio recall (109). Diabetes insipidus, a disorder characterized by a mutation in the vasopressin prohormone that leads to lower plasma levels of AVP, is associated with decreased performance on a test of verbal memory in humans (110). Although elevations of AVP in the bloodstream can lower detection of auditory signals, once those signals are detected, AVP can enhance recall of information provided in those signals.

### AVP and processing of taste information

#### AVP and conditioned taste avoidance

A key to understanding the evolution of taste aversion in mammals and its link to social behavior has been provided by studies of gustatory learning in *Caenorhabditis elegans*, a nematode that utilizes chemoattraction to locate low salt environments (111). Modulation of this ability occurs through the action of a vasopressin/oxytocin-like neuropeptide, nematocin. When exposed to preferred low salt environments in the absence of food, worms with a null mutation for nematocin or its receptor are not able to learn to avoid these environments (111). Individuals lacking nematocin or its receptors also exhibit deficiencies in male mating behavior and other forms of social behavior due to altered activity of mechanosensory neurons (112). These findings implicate the ancient neuropeptide, nematocin, in the integration of sensory input into complex motor output in the model organism *C. elegans*. This link between AVP, taste, and social behavior seems to be preserved in more complex organisms like birds and mammals. In the zebra finch (*Taenioypygia guttata*), intraseptal infusion of a V1 antagonist reduces gregariousness and increases the latency to feed in the presence of a novel stimulus (113). However, in mammals, it is unclear whether the effects of AVP on taste also modify social behaviors.

Arginine vasopressin within the bloodstream may alter the processing of taste in mammals through a mechanism similar to the one described above for auditory learning. By altering arousal through activation of the sympathetic nervous system, AVP improves an animal’s ability to learn to avoid an aversive stimulus. In rats, taste aversion to saccharine lasts longer if administration of desglycinamide-lysine vasopressin occurs prior to pairing of the taste of saccharine with nausea induced by injections of LiCl (114). Other studies, however, have demonstrated the opposite relationship with AVP injections leading to faster extinction of learned responses. The timing of AVP administration appears to be critical because the learned association disappears more rapidly if AVP is administered after the acquisition process, regardless of whether the AVP is administered peripherally or centrally (115–117). However, dosage of AVP does play a critical role because high doses are capable of inducing taste aversion without administration of LiCl following consumption of a sucrose solution (117). Although peripheral AVP injections produce similar effects as central AVP infusions, this effect still seems to result in part from the release of AVP from the neurons of the PVN. AVP is released in the PVN during extinction of taste avoidance responses in rats that have not been deprived of water as part of the testing protocol (118). Because radioimmunoassay shows no changes in AVP content in brain areas that have been associated with conditioned taste avoidance such as the medial septum, LS, insular cortex, or MA, this increase in AVP content in the PVN is most likely related to the osmoregulatory properties of AVP in the PVN (118). However, future studies may clarify the role of neural AVP in regulating taste aversion. Taken together, these data indicate that AVP can either improve sensory learning or hasten extinction of learned responses depending on the timing of AVP release.

#### AVP and modulation of taste

**Figure 2:**
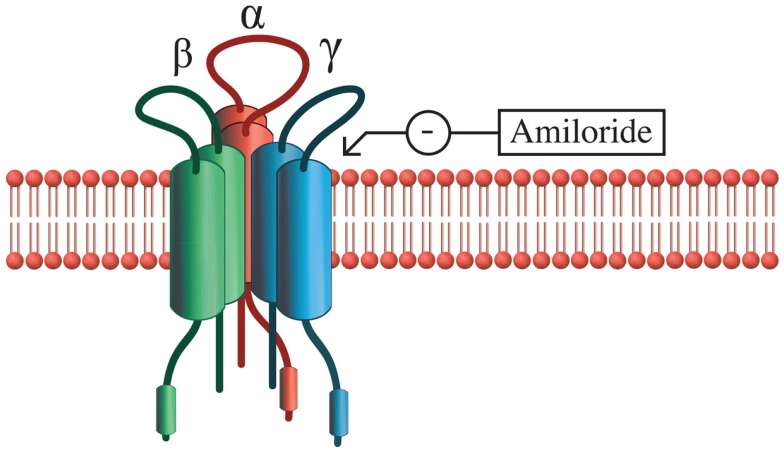
**Epithelial Na^+^ channel structure**. ENaCs are composed of three subunits: α, β, and γ, and transport salt in a variety of epithelial tissues in the body. The α subunit alone is capable of functioning as a sodium transporter, but the addition of β and γ subunits increase the efficiency of this process. Sodium transport activity of ENaCs can be blocked with amiloride or enhanced with AVP.

One molecular mechanism that AVP may use to modify taste is through modulation of the activity of epithelial sodium ion channels (ENaCs) that regulate salty and sour tastes [Figure 2; (119)]. ENaCs are found in the renal collecting duct, urinary bladder, lung, and taste buds and consist of three subunits that form a pore that selectively permits sodium to cross the membrane [reviewed in Ref. (120)]. In fungiform taste cells of hamsters, AVP increases sodium ion currents in ENaCs, indicating that the threshold for stimulation of these cells is being lowered (121). Therefore, AVP in the bloodstream may increase sensitivity to salty or sour tastes through its action on the ENaCs in taste cells. Under conditions where blood volume is low, this response may be part of a homeostatic mechanism to increase water retention in the blood by enhancing the palatability and consumption of salty food (121, 122). Although high doses of AVP appear to inhibit salt intake by making animals feel ill, central administration of lower doses of AVP stimulate salt intake whereas V_1_ receptor antagonists inhibit salt intake (123). At the neural level, ENaCs also appear to be involved in regulating salt intake because they are co-localized with AVP in the magnocellular neurons of the SON and PVN, cells that assist with osmoregulation (124). Interestingly, these neural sodium channels are similar to ENaCs of the tongue in that both exhibit sensitivity to amiloride, a potassium-sparing diuretic that inhibits taste responses (124). In the brain, ENaCs may act as sensors of salt concentrations on the cells of the PVN and SON and lead to alterations in neural firing rates in response to fluctuations in salt concentration in the cerebrospinal fluid (124). Although it is unknown how ENaC activation by AVP may alter complex social behavior in mammals, these data indicate that behavioral responses that lead to release of AVP from the PVN and SON may also increase sensitivity to taste.

Disruption of taste sensitivity may be an important clue in diagnosing diseases that are accompanied by AVP dysregulation, such as syndrome of inappropriate ADH secretion (SIADH) (81, 125). In patients with SIADH related to lung cancer, the excess AVP alters taste perception (81, 126, 127). In patients with low serum sodium levels due to the effects of AVP on blood osmolality, correction of these levels led to improvement of taste function (81, 126, 127).

### AVP and visual processing

Although visual information feeds into many brain areas that contain AVP, such as the BNST and LS (128, 129), the effect of AVP on processing of visual information has received little attention. Comparative analysis of visual opsin sequences in a variety of vertebrate species indicates that the visual opsins and AVP receptors are found in a shared genomic region that was duplicated twice during vertebrate evolution (130). However, the behavioral significance of the linkage between these sequences has not been examined. In chickens, a visual opsin has been co-localized with AVT in the neurons of the PVN, SON, and BNST (131). Although the function of opsins outside of the eye is unknown, these pigments may allow these neurons to respond to light and assist with coordination of circadian rhythmicity in behavior (131).

Connections between AVP and visual processing have been made in studies of visual learning, although these studies have focused more on AVP enhancement of sensory learning instead of AVP alteration of visual processing [e.g., Ref. (132, 133)]. In non-mammalian species, however, it has been demonstrated that AVT, a homolog of AVP, stimulates interest in visual cues associated with reproduction in rough skin newts (134). Additional studies in mammalian species that rely on visual cues during social interactions will help to clarify the role of AVP in visual processing.

## Conclusion and Future Directions

A social animal is bombarded with sensory cues throughout his or her lifetime. Even at birth, animals are usually attracted to the odors of their mothers but avoid unfamiliar odors. These and similar sensory cues are channeled through the central nervous system of the animal leading to output in the form of the social behaviors that are necessary for that individual’s survival. In social rodents who heavily utilize their olfactory senses to distinguish threat from other environmental stimuli, AVP has been localized within the olfactory bulb itself and within the targets of the olfactory pathways. Therefore, as AVP neurons of the main and accessory olfactory systems are activated, they may modulate this olfactory input and direct it to the appropriate sites in the brain that regulate behavioral output. In addition, olfactory pathways also send information to the magnocellular neurons of the PVN and SON, which release AVP into the bloodstream. Additional research may clarify the degree of interplay between these two systems. The relationships between AVP in the olfactory system and other neurotransmitter systems and other sensory systems also should be explored in greater detail.

Although evidence also indicates that a relationship exists between AVP and other sensory modalities, these relationships have not been studied as extensively as the relationship between AVP and olfactory processing. Because most studies examining the role of AVP in sensory processing have been performed in rodents that use olfaction as the primary sense, other sensory modalities have received less attention. At this time, it is not known whether AVP in brain areas associated with mammalian visual, taste, or auditory processing shows the same level of plasticity in response to social or hormonal cues that shape behavior. It is also unknown whether manipulations of AVP can produce broad alterations in sensory processing in a variety of modalities in mammalian species with different social systems. How an animal is able to filter these social cues and make complex behavioral decisions needs to be explored in additional detail. However, it is clear that AVP modulates sensory processing through a variety of neural and hormonal pathways that are critical for the expression of species-typical social behavior.

Arginine vasopressin may be essential for integration of sensory input during complex forms of social behavior in mammals. As described earlier in this paper, fMRI has been used to assess neural activation in maternal rats presented with the threat of a male intruder (27). When a V1a antagonist is administered prior to exposure to the intruder, the gustatory cortex and olfactory areas of the brain show enhanced BOLD responses in females (27). This finding indicates that AVP is involved in regulating multiple sensory responses during complex social interactions, but should be explored under other social contexts and in additional species.

Studies using peripheral and central injections of AVP have demonstrated that the hormonal effects of AVP can shape gustatory, visual, and auditory learning through its effects on general levels of arousal. In addition, levels of AVP within the bloodstream can influence sensory processing in the ear and tongue through modulation of sodium channels. Within the ear, excess AVP has been associated with impaired hearing due to excess endolymph accumulation. In the tongue, AVP appears to modulate the activity of sodium channels known as ENaCs. Whether AVP shows similar ability to regulate visual signal transduction in a similar way has yet to be explored.

The apparent consequence of this fine-tuning of sensory input is that an animal is able to display appropriate social behavior in response to particular environmental stimuli. Animals with null mutations in vasopressin or its receptors demonstrate sensory deficits that appear to be potentially correlated with deficits in social behavior. In addition, the plasticity in AVP pathways during development that has been demonstrated in a variety of rodent species (2–4, 8, 135–137) may affect social behavior through alterations in the processing of sensory signals. However, the impact of developmental plasticity in AVP on sensory processing in multiple modalities has not yet been explored.

In humans, attempts have been made to link AVP with disorders that affect social behavior and involve sensory processing issues such as autism and schizophrenia [reviewed in Ref. (138)]. Because it is impossible to directly manipulate AVP or its receptors within specific brain areas in humans, the relationship between AVP and behavioral disorders has been assessed by correlating plasma AVP levels or V1a receptor promoter polymorphisms with behaviors associated with these disorders. Although the relationship between AVP and social behavior disorders has been difficult to establish in males, plasma levels of AVP have been linked to severity of psychosis in women with schizophrenia (139). Similarly, in girls with autism, plasma levels of AVP have been linked to the intensity of repetitive behaviors (140). Genetic linkages between AVP and the likelihood of developing a disorder of social behavior have also been identified in humans. A specific genetic polymorphism in the V1a receptor promoter was associated with increased susceptibility of psychopathology in children that had been exposed to war (141). The latter finding suggests that although certain V1a genotypes may predispose an individual to developing a disorder related to social behavior, exposure to environmental stressors also plays a role in the manifestation of the disorder. Therefore, it is possible that early social experiences may create life-long changes in the way that an individual perceives sensory cues in the surrounding world.

## Conflict of Interest Statement

The authors declare that the research was conducted in the absence of any commercial or financial relationships that could be construed as a potential conflict of interest.
